# Assessment of Clayey Peloid Formulations Prior to Clinical Use in Equine Rehabilitation

**DOI:** 10.3390/ijerph17103365

**Published:** 2020-05-12

**Authors:** Carla Marina Bastos, Fernando Rocha, Ângela Cerqueira, Denise Terroso, Cristina Sequeira, Paula Tilley

**Affiliations:** 1 GeoBioTec Research Centre, Department of Geosciences, University of Aveiro, 3810-193 Aveiro, Portugal; tavares.rocha@ua.pt (F.R.); angelamcerqueira@ua.pt (Â.C.); laraterroso@ua.pt (D.T.); csequeira@ua.pt (C.S.); 2 Exatronic, Lda, 3800-373 Aveiro, Portugal; 3 CIISA – Centre for Interdisciplinary Research in animal Health, Faculty of Veterinary Medicine, University of Lisbon, 1300-477 Lisbon, Portugal; paulatilley@fmv.ulisboa.pt

**Keywords:** ethnoveterinary, pelotherapy, healing clays, quality control, equine limb injuries

## Abstract

Clays are natural ingredients used to prepare therapeutic cataplasms suitable for topical application. The knowledge about these formulations and their preparations to be applied on humans and animals has been orally transmitted since ancient times. Several empirical methods using clays have demonstrated fast and effective results in the reduction of the inflammatory response and the formation of edemas in horse limbs. The use of traditional and alternative medicine, such as pelotherapy, is now becoming more popular in veterinarian medical practice, alone or combined with other therapies in horse muscle and tendon rehabilitation. This study characterizes the use of commercial equine clays and an old therapeutic clay cataplasm formulation, using acetic acid, to treat tendon injuries in horses. This work might contribute to a major database characterization of clays used empirically on equine health, the potential of dermal absorption, the risks of exposure to some toxic elements, and safety assessment for these formulations. The present study was carried out to characterize the suitability of four commercial equine clays (Group II) and a protocoled healing mixture: “clay acetic acid cataplasm”, (Group III), to treat tendon injuries in horses. In this mixture, three conventional “green” clays (Group I) without any mineralogical specificity were used and blended with acetic acid. The mineralogical composition was determined through X-ray powder diffraction and X-ray fluorescence data. To determine the performance of the samples, cooling kinetics, oil absorption, expandability, and specific surface area were measured. According to the mineralogical composition, Group I was mainly composed of carbonates and silicates, while Group II was much richer in silicates with the main clay minerals kaolinite and illite. Group II exhibited the highest values for As, Pb, Cr, Ni, and Zn, considered potentially toxic. Both groups showed low cation exchange capacities and exchanged mainly Ca^2+^, with the exception of VET.1 and VET.7, which also highlight Na^+^, and VET.5 and VET.6, which have K^+^ as an exchangeable main cation. The addition of acetic acid (Group III) does not reveal any significant chemical changes. The results confirm that both clay groups are adequate for the therapeutic propose. They have good plastic properties (skin adherence), good oil absorptive capabilities (cleaning), and exchange an essential physiological element, calcium. Group II has prior industrial preparation, which is probably why it showed better results. Group I presented lower heat retention capacity and higher abrasiveness, which could be improved using cosmetic additives. The clinical benefit of the “clay acetic acid cataplasm” (Group III) could be the systemic anti-inflammatory effect established by the acetic acid.

## 1. Introduction

It is well documented that clayey formulations have been important resources for human and animal health care, because of their therapeutic and curative properties, since the first records in history [1,2]. 

Several traditional veterinary practices use zootherapeutic resources in the health care of domestic animals, the medicinal value of which maintains its relevance in ethnoveterinary medicine (EVM), the scientific term for traditional animal health care [3]. Clay minerals and their healing powers in wild animals are well documented by the practice of eating clay (geophagy) for detoxification of the body and alleviation of gastrointestinal infections and are now being rediscovered [4]. Kaolin and smectitic clays are commonly used in animal nutrition as growth promoters and supplements for the treatment of gastrointestinal disturbances. The introduction of kaolin clay, as feed additive, to treat foals with “heat” diarrhea, caused by disturbances in the intestinal osmotic balance of the young horses succeeds well as an absorbent and as an anticaking agent, alleviating the severity and duration of foal heat diarrhea [5].

The veterinary industry responds to the equine market with specific clayey products, promoting them by their pharmacological effects. The clayey products tailored for lameness injury prevention are relevant indicators for the evaluation of the therapeutic impact of pelotherapy in equine health and product procurement. 

The clays’ efficacy for lameness or other musculoskeletal injuries on horses is free of regulatory compliance. Most of these clayey products are designed accordingly with requirements and specifications supported by specialized equine technicians.

The use of pelotherapy as a therapeutic modality is scientifically little explored in equine health, despite its recognition as a valid non-invasive therapeutic option. 

There are a few veterinarian databases, designed to search for relevant studies and clinical trials reported by researchers, such as PubMed and IVIS Quick-Links. The AVMA Animal Health Studies Database (www.avma.org/findvetstudies) allows submission and search of studies for health care issues in dogs, cats, horses, or other animals. Using “horse” and “equine” as a keyword we did not find any issue related to the use of clays in equine rehabilitation.

Clay minerals are widely used in pharmaceutical formulations as excipients and because of their biological activities [6] and are used in cosmetics because of their physical and physical–chemical properties such as adsorption capacity, specific surface area, swelling capacity, and reactivity to acids [7]. 

Williams and Haydel (2010) made the distinction between “healing clays” and “antibacterial clays”, which may cure several diseases only by their unique physical and chemical properties (e.g., high absorbance, surface area, heat capacity, exchange capacity, etc.) or by killing pathogenic bacteria [8].

The absorptive capabilities of clays have been explored in a variety of cosmetic and pharmaceutical formulations and as a contributor to the healing of diseases, as well as for their cation exchange capacity and extremely fine particle size, which are important properties for removing oils, secretions, toxins, and contaminants from the skin. Cation exchange experiments showed that the antibacterial component of the clay can be moved, implying the presence of exchangeable cations in the antibacterial process [8]. Studies made on a natural clay from the Colombian Amazon and compared to the standard reference of smectite and kaolinite showed chemical interactions that are detrimental to bacteria by absorbing nutrients (e.g., Mg, P) and by toxic metal supply (e.g., Al) [9].

Humans and equine athletes share acute and chronic tendon injuries as the most common orthopedic affections, having similar structural (reparation) and functional (regeneration) recovery process. [10]. 

Ca, P, K, and S play a pivotal role in both the growth and the degeneration of the collagenous bone–cartilage interface of articulating joints demonstrated on equine osteoarthritic lesions (metacarpophalangeal joint) by detecting variations of elemental presence, using Synchrotron radiation micro X-ray fluorescence analysis [11].

The conservative veterinary therapy protocols include the same considerations as the human medicine protocols for orthopedic affections: cold applications, pressure-supporting bandages, controlled exercise, medicines to be injected, electrotherapy sessions, electromagnetic stimulation, ultrasound and laser therapy, or in an extreme clinical recommendation, surgical therapy [10]. Intra-articular (IA) administration of drugs in the treatment of musculoskeletal injuries has the objective of directing the drug delivery to the affected tissues and is commonly used by veterinarians and by Medical Physical Rehabilitation specialists. The use of corticosteroids or nonsteroidal anti-inflammatory drugs is common in racehorses and has become a problem for veterinarians due to the fact that the medicine could be masking a possible musculoskeletal condition and may contribute to injuries during competition [10,12]. Although there are a significant number of nonsteroidal anti-inflammatory drug (NSAID) formulations designed for the treatment of muscle and tendon traumatic conditions in human beings, when compared with the same problem in equine clinical practice, these formulations are more commonly used in horses. In vitro studies to evaluate and compare the penetration of diclofenac, a common NSAID designed for human application, revealed a significantly lower penetration through horse skin [13].

Complementary and alternative medicine (CAM) gained good acceptance in human medicine, mainly in the treatment of musculoskeletal pathologies and is now getting some popularity in veterinary medicine [14], therefore, regenerative therapies in horses may have applications for future human medicine and vice versa [10]. The initial interest and positive opinion on complementary alternative veterinary medicine (CAVM) started amongst horse owners. Most of them applied CAM therapies without the previous knowledge of their veterinarian, mainly to avoid possible conflict and fearing that their veterinarian might not want to continue providing veterinary care for their horse [14]. Some of this CAVM was supported by traditional Chinese veterinary medicine (TCVM), using acupuncture physiology to treat pain [15]. 

There is a lack of dissemination of traditional therapeutic procedures or rehabilitation programs using pelotherapy by key users (e.g., equine owners, equine trainers, equine veterinary, and industry) in the research field. 

Equine rehabilitation programs must be designed with the previous identification of the risk factors that could predispose to musculoskeletal injury, considering the phases of healing, the rehabilitation goals, and the techniques used for acute injuries in horses [16]. 

The use of clays in these rehabilitation programs should fulfill the requirements regarding their safety and stability and should preferably be subjected to pre-market approval. 

In this work, we characterize the mineralogical composition and technical performance of equine peloids used in prevention and rehabilitation programs. The main goals are contribution to the establishment of veterinarian clay therapeutic criteria, disclosure of the protocoled healing mixture: “clay acetic cataplasm”, and to contribute to ethnoveterinary scientific databases.

## 2. Materials and Methods 

### 2.1. Data Preparation

For this study, we selected seven commercial clays routinely used in veterinary medicine and suggested by the CIISA—Center for Interdisciplinary Research in Animal Health (University of Lisbon, Portugal) for the treatment of equine musculoskeletal injuries, namely front- and hind-limb tendon and ligament injuries. VET.1, VET.5, VET.6, and VET.7 are four industrial pasty clays sold in the market as an equine clay treatment to be applied in a thick layer against the lay of the hair, after intensive exercise or a competition. VET.2, VET.3, and VET.4 are natural “green” clays, sold for human application and with no specific usage recommendations.

The protocol performed by CIISA for the treatment of horse musculoskeletal limb injuries proposes a 1:10 acetic acid (AA) and piped water solution with the necessary proportion of dried “green” clay. The CIISA protocoled solution was prepared with food acetic acid, pH 2.7 at 25 °C resulting in a solution (1:10) with pH of 2.9 at 25 °C. This protocol solution is only applied on VET.2, VET.3, and VET.4. This resulting cataplasm must ensure adhesive proprieties when applied to the injured area. After this, the injured area is wrapped in cling film, which acts as a thermal adjuvant, prolonging the therapeutic effect of the clays. The animal is then supervised by the clinical therapist, who decides when the clay effect is assured. We compared the commercial clays’ results with the protocoled healing clay and acetic acid mixture. 

Clays were distributed in three groups (Table 1), Group I and Group II according to their commercial purpose, and Group III for the protocoled healing mixture. All samples were dried at 50 °C, with no previous treatment, and maintained in closed containers at room temperature. For the preparation of the protocoled mixture, 10 g of the Group I clays was dispersed in 10 mL of acetic acid solution (1:10) and left to rest for 24 hours. These samples, VET.2AA, VET.3AA, and VET.4AA, were also dried at 50 °C. The pH value of the samples was measured with a HANNA HI 9126 pH meter, previously calibrated with standards (Titisol standard solutions) at pH 4 and pH 7 with an accuracy of ±0.05. 

### 2.2. Mineralogical, Chemical, and Technological Analysis

The mineralogical analysis was carried out by X-ray diffraction (XRD) analysis, using a Philips/Panalytical X’Pert-Pro MPD, Kα Cu (α = 1.5405 Å) radiation, with 0.02° 2θ s^−1^ steps in goniometer speed. For the preparation of preferentially oriented aggregates of the clay (<2 µm fraction), a suspension was placed on a glass slide and air dried. XRD scans were run on this air-dried glass slide, and afterward a glycerol saturation and a final heat treatment at 500 °C were carried out [17]. The semi-quantitative identification of the principal clay minerals was obtained by measuring peak areas of the basal reflections, considering the full width at half maximum and then weighted by empirically estimated factors [17,18]. 

The particle size distribution of these clays was determined by an X-ray beam particle size analyzer (Micromeritics Sedigraph III Plus). The samples were dried and washed with distilled water, resting for 24 hours to ensure the separation between all particles. Then, the samples were sieved (106 µm) and dried. The dried sample was gently disaggregated, and the uniformity was ensured by quartile distribution. After that, 80 mL of sodium hexametaphosphate was added to 5.8 g of each sample and left to stand for 8 hours with magnetic stirring. At the end, the sample was sieved again (106 µm) and submitted to ultrasound for 40 s before the equipment measurement step.

The chemical composition of the commercial clays was assessed by X-ray fluorescence (XRF) using a Panalytical AX-IOS PW 4400/40. Loss on ignition (LOI) was also assessed by heating 1 g of the sample at 1000 °C for 1 hour in a furnace.

Abrasiveness was measured with an Einlehner AT-100 apparatus [19,20], and Atterberg limits were assessed using Casagrande Shell to obtain the liquid limit and using molding rolls in a glass plate for the plastic limit [20,21]. The plasticity index was calculated in accordance with the Portuguese standard, NP 143-1969. The expansion index test was performed by the standard LNEC E200-1967, Portuguese edition for ASTM (2008) to measure the swelling capacity of the samples when absorbing distilled water [20].

Samples were heated to 60 °C and the heat diffusiveness was assessed by a dual-channel thermometer, Lutron TM-9064. The range of time values was measured between 60 and 29 °C. Linseed oil was used to measure the oil absorption capacity of the clays. Fifteen grams of dry clay was weighed together with an amount of linseed oil. In a glass plate, linseed oil was slowly added, drop by drop, until it was possible to achieve the consistency necessary to obtain a solid roll of clay. The remaining oil and the clay roll were weighted for the oil absorption calculation.

The cation exchange capacity (CEC) was estimated by the ammonium acetate method, and the exchangeable cations (Na^+^, K^+^, Mg^2+^, and Ca^2+^) were determined by an atomic absorption spectrophotometer [22]. Specific surface area (SSA) was estimated by BET analysis—Gemini II 2370.

## 3. Results

### 3.1. Mineralogical and Chemical Characterization

#### 3.1.1. Grain Size Distribution and Mineralogical Composition

**Figure 1:**
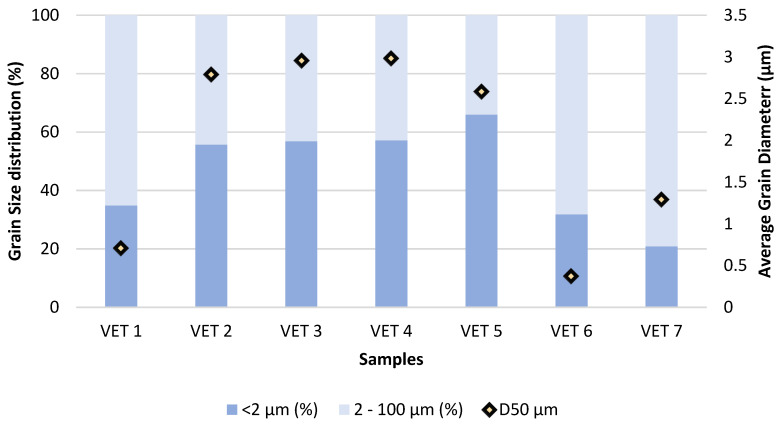
Grain size distribution of the Group I and Group II samples.

The results from the particle size distribution of the samples are shown in Figure 1. Group I samples contained more than 55% of fine fraction content, with an average diameter of ~3 µm. In Group II, VET.1, VET.6, and VET.7 had around 65%, 68%, and 79%, respectively, of particles sized between 2 and 100 µm, and different average diameters, VET.1 and VET.6 had a D_50_ of 0.708 and 0.373 µm, and VET.7 had a D_50_ of 1.292 µm. For the VET.5 sample, the granulometry size distribution was as in Group I samples, 66% of fine fraction content and an average diameter of ~3 µm. 

**Figure 2:**
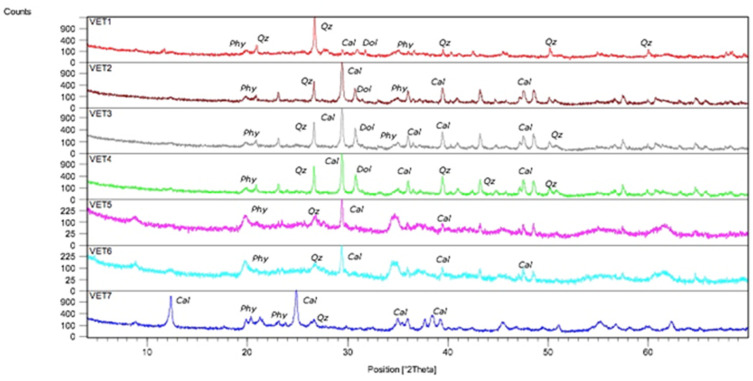
X-ray diffraction (Cal: calcite; Qz: quartz; Dol: dolomite; Phy: phyllosilicates).

**Figure 3:**
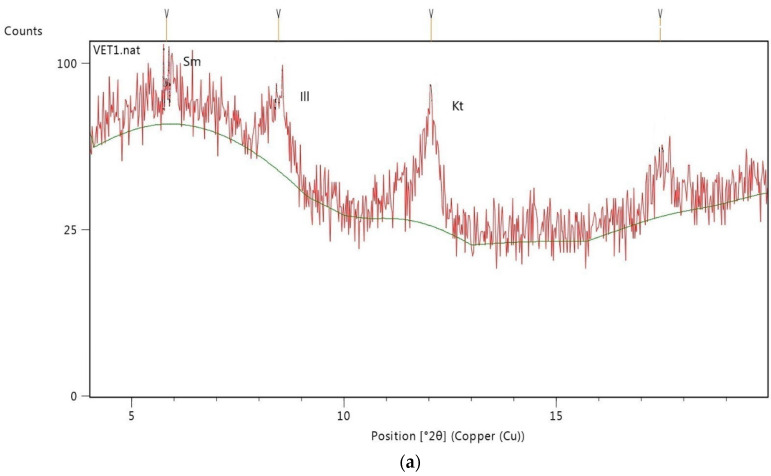

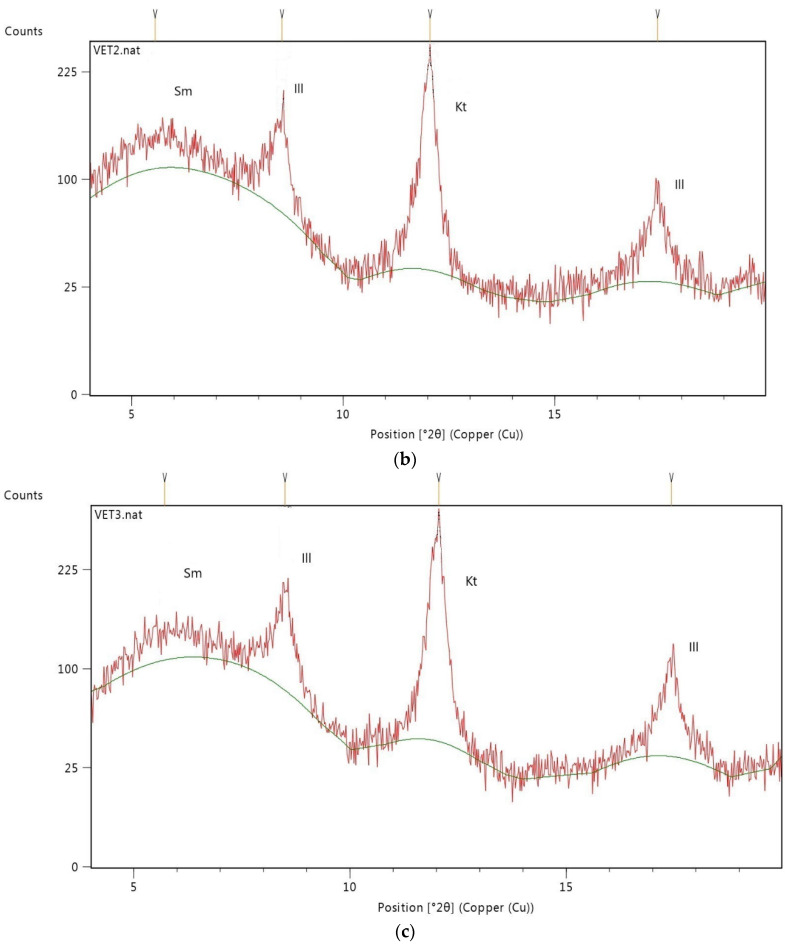

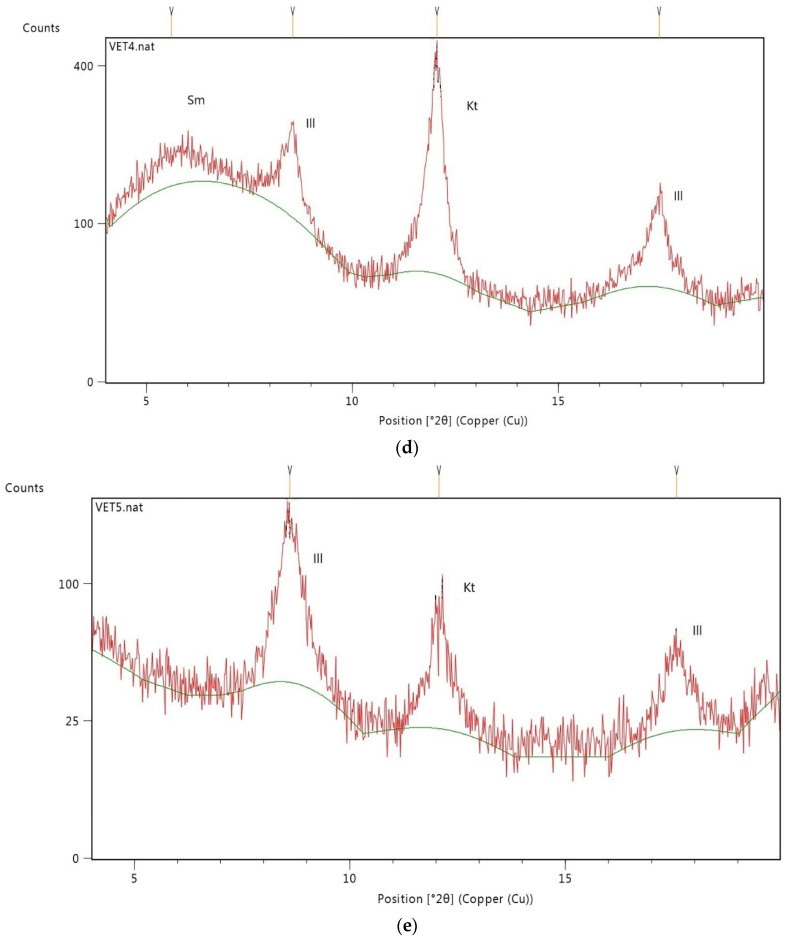

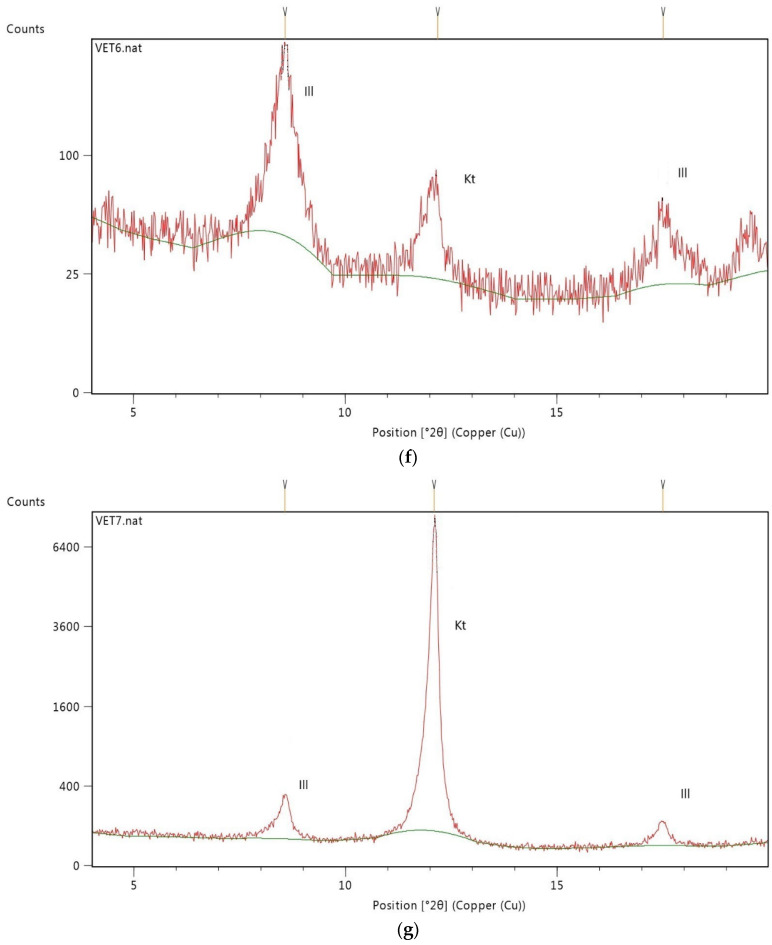
X-ray diffraction patterns of air-dried oriented aggregates (**a**) VET.1; (**b**) VET.2; (**c**) VET.3; (**d**) VET.4; (**e**) VET.5; (**f**) VET.6; (**g**) VET.7. (Ill: illite; Sm: smectite; Kt: kaolinite).

The mineralogical composition of the Group I and Group II clays is reported in Table 2. All samples were polymineralic (Figure 2) and exhibited differences in mineralogical composition. Considering the average for each group, we can classify Group I (n = 3) mineralogically as being composed by carbonates (calcite and dolomite) and silicates (quartz and phyllosilicates/clay minerals), while Group II is much richer in silicates, with a pronounced increase in phyllosilicates/clay minerals. The main clay minerals (Figure 3) are kaolinite (25%) followed by illite (7%) in Group I, and illite (68%) followed by kaolinite (2%) in Group II, except for VET.7 (67% kaolinite and 28% illite). 

#### 3.1.2. Chemical Composition

The content of major and minor chemical elements is shown in Table 3. Differences in chemical composition were in accordance with those detected in the mineralogical composition; Group I (the more carbonated) was richer in CaO (27%) while Group II was richer in SiO_2_ (44%) as well as in Al_2_O_3_, Fe_2_O_3_, and K_2_O.

Considering the chemical elements that are potentially toxic and not allowed in care products (Regulation (EC) 1223/2009), the Group II exhibited the highest values for As, Pb, Cr, Ni, and Zn when compared with Group I, for human usage. 

Group II shows high levels of Pb and As, however, it is difficult to estimate equine exposure and the health risks associated. 

### 3.2. Physical and Technological Characterization

Both groups show low cation exchange capacities and exchange mainly Ca^2+^, with the exception of VET.1 and VET.7, which highlight also Na^+^; and VET.5 and VET.6, which have K^+^, as an exchangeable main cation (Table 4 and Table 5). They have good plasticity, which is necessary to ensure adhesiveness to the skin. For Group I and VET.1, an abrasiveness action is expected when in contact with the skin surface, but the impact depends on the skin condition of the animal and horsehair protection. 

## 4. Discussion

These two groups of clays recommended by the CIISA—Center for Interdisciplinary Research in Animal Health, University of Lisbon, for the treatment of horse musculoskeletal injuries, have different compositional and textural characteristics.

Considering application and topical use characteristics (Table 6), taking as reference recommended values published by several authors [8,19,20,22,23,24,25,26,27,28,29,30,31,32,33,34], the samples studied show good plastic properties, which are necessary for skin adherence; good oil absorptive capabilities, which is important to clean the skin from impurities or wound secretions; and Group II has a good heat retention capacity, important when it is necessary to heat the cataplasm, to active the blood circulation. The abrasiveness of Group I clays, which can cause unnecessary rubbing on the animal´s injured skin, should be smoothed.

Calcium is an essential element that is important for the growth and regeneration of collagenous bone-cartilage and this may explain the traditionally used “green” carbonated clay for the acetic acid cataplasm formulation. Despite As and Pb being technically avoidable above 0.5 and 2.0 mg/kg [25], respectively, the fact that they are above the risk limit that is internationally accepted for pharmaceutical formulations and cosmetics applied to human beings, and that several factors should be considered in transdermal penetration for humans and for horses, the same formulation may have different efficacies and safety profiles when used in species for which they were not developed [13]. Group II are clays that were industrially developed specifically for equine usage.

The addition of acetic acid (Group III) does not reveal any significant chemical changes when compared with Group I, apart from the pH that becomes more alkaline (closer to 8). All samples have a pH around 7 and 8. Organic acids (e.g., acetic acid) are usually used in food as natural preservatives and antibacterial agents. The manipulation of clay minerals to eliminate clinical and environmental bacteria is very common and the investigation of the antibacterial properties of natural clays has taken a new approach [8,26,27,28], where the possibility of incorporating bactericidal properties into clays may also be activated by the use of an acid solution in the treatment [27]. Some studies reveal the strong antibacterial effect of acetic acid combined with silver nanoparticles (AgNPS), where the release of Ag^+^ responsible for the antibacterial activity increased by the addition of acetic acid [28]. 

The obtained data, when analyzed in comparison to reference values, allowed us to consider that both groups are adequate for therapeutic proposes, such as the treatment of horse musculoskeletal injuries, but Group II shows the best characteristics. Group II, having a prior industrial preparation, is technologically adapted for use in horses. The use of additives and preservatives in their preparation may be the reason why they are less abrasive and toxic.

The establishment of a health database considering ethnoveterinary medicine studies may enrich the equine health databases, useful to institutions, veterinarians, animal owners, and also providing guidelines for the investigation of new therapies and for scientific evidence findings.

## 5. Conclusions

The potential benefit of using therapeutic clays in equine lameness injuries is to minimize the side-effects associated with oral and intra-articular administration of anti-inflammatory medicines and to sustain a local release of therapeutic elements. This study can also be considered as a contribution to a major database of clays used for animal topical application, to the awakening of traditional and ancestral methodologies in healing clay preparations, and also to the knowledge about the contribution of these clays in the rehabilitation programs developed by veterinarians. 

Through this study we tried to address the knowledge gaps about the mineralogical and chemical composition of clays used for equine peloid preparation and assessed their technical performance. This research assured that the studied clays fulfill the safety and stability requirements for these rehabilitation programs, thus, enabling them to be submitted for pre-market technical and legal approval process.

The main limitation of this study was the impossibility to correlate CIISA therapeutic formulation results with results obtained with specific equine commercial clays. Further work is needed to compare the antibacterial effectiveness of this protocol with other mud cataplasm protocols applied to horses and humans. This would be important and necessary for the establishment of healing criteria for veterinary clays.

Most of the thermal spas around the world recommend their own mud baths or local mud cataplasm applications, as they recognize therapeutic results through their anti-inflammatory, analgesic, and antiseptic effects on musculoskeletal and dermatologic pathologies, which are increasingly supported by clinical trials. The efficacy of the candidate clays to be used in veterinary pelotherapy should be evaluated and compared with human pelotherapy results and supported by clinical trials. 

The safety and regulatory compliance of these products should also be a priority. The identification of unwanted trace elements or toxic substances based on the raw material source (natural or synthetic) should be a determinant for market surveillance of the appropriate limits expected in these natural products.

## Figures and Tables

**Table 1:** Sample identification.

Group	Samples	Type	Commercial Purpose
I	VET.2	Powder	Human dermal application
	VET.3	Powder	Human dermal application
	VET.4	Powder	Human dermal application
II	VET.1	Paste	Equine dermal application
	VET.5	Paste	Equine dermal application
	VET.6	Paste	Equine dermal application
	VET.7	Paste	Equine dermal application
III ^1^	VET.2AA	Paste	Protocoled healing mixture
	VET.3AA	Paste	Protocoled healing mixture
	VET.4AA	Paste	Protocoled healing mixture

^1^ CIISA—Center for Interdisciplinary Research in Animal Health protocol.

**Table 2:** Mineralogical composition (%).

		Total Sample (%)				Clay Minerals (%)		
Group	Samples	Qz	Hal	Calc	Dol	Sm	Kt	Ill
I	VET.2	6	-	47	13	<1	29	5
	VET.3	9	-	43	21	<1	21	6
	VET.4	10	-	39	17	<1	24	10
II	VET.1	44	2	-	-	1	3	50
	VET.5	8	-	15	-	-	4	73
	VET.6	3	-	16	-	-	1	80
	VET.7	5	-	-	-	-	67	28
III	VET.2AA	7	-	52	16	<1	19	6
	VET.3AA	14	-	56	10	<1	17	3
	VET.4AA	11	-	58	11	<1	17	3

Qz = quartz; Hal = halite; Calc = calcite; Dol = dolomite; Sm = smectite; Kt = kaolinite; Ill = illite.

**Table 3:** Major and minor element composition of the samples. LOI = loss on ignition.

Samples		Group I			Group III			Group II			
		VET.2	VET.3	VET.4	VET.2 AA	VET.3 AA	VET.4 AA	VET.1	VET.5	VET.6	VET.7
Major elements (wt.%)	SiO_2_	24.424	26.272	24.924	25.457	25.009	25.499	46.078	41.723	41.110	46.206
	Al_2_O_3_	10.508	11.147	10.488	11.062	10.906	11.120	15.417	18.195	17.980	35.098
	Fe_2_O_3_	2.597	2.957	2.664	2.885	2.721	2.916	6.185	6.423	6.354	0.778
	MgO	3.364	2.742	3.423	2.334	2.535	2.415	2.626	3.129	3.271	0.427
	CaO	26.859	25.976	27.391	28.636	27.071	28.811	4.233	4.901	5.841	0.049
	Na_2_O	0.096	0.083	0.082	0.093	0.084	0.088	3.292	0.146	0.147	0.781
	K_2_O	1.568	1.649	1.585	1.793	1.692	1.788	2.976	6.111	6.184	1.831
	TiO_2_	0.328	0.363	0.342	0.352	0.348	0.366	0.824	0.629	0.649	0.023
	P_2_O_5_	0.046	0.048	0.041	0.052	0.047	0.046	0.196	0.166	0.233	0.144
	SO_3_	1.295	1.265	1.219	1.081	0.998	1.068	2.239	0.323	0.029	0.100
	LOI	28.65	27.20	27.65	25.96	28.270	25.550	13.83	17.85	17.80	14.3
Minor elements (ppm)	As *	●	●	●	●	●	●	17	24.6	21.5	8.2
	Cd *	●	●	●	●	●	●	●	●	●	●
	Pb *	13.7	14.4	12.8	16.1	14.8	16.1	31.6	32.2	31.8	21.6
	Cr	51.9	57.4	53.4	54.6	52.3	54.8	130	68.5	66.7	4.2
	Cu	15.4	8.1	15.0	8.4	11.6	11.6	14	22.2	24.4	29.5
	Ni	16.0	19.7	16.0	17.7	15.4	16.1	33.6	29.0	29.3	5.0
	Zn	24.3	28.2	26.9	24.3	23.0	23.2	95.2	120	140	22.3
	Ba	110	120	150	160	150	110	250	280	200	150
	Co	●	7.5	4.9	5.5	6.1	4.8	14.7	10.2	11.7	●
	Sr	230	220	200	240	220	240	210	180	280	160
	V	59.7	74.5	72.4	57.2	56.4	57.7	100	72.2	78.1	5.4
	Sb *	●	●	●	●	●	●	●	●	●	●
	Sc	19.8	18.6	17.1	20.6	17.9	21.0	10.9	10.1	11.2	●

● Not determined; * Potentially toxic elements.

**Table 4:** Main physical and technological properties of the studied samples.

Group	Samples	P.I. (%)	A.I. (g/m^2^)	C.E.C. (meq/100)	E.C. (mg/L)			
					Na	Mg	K	Ca
I	VET.2	25	142.85	7	1.67	25.28	9.63	730.81
	VET.3	26	236.77	7	1.79	27.76	7.45	734.50
	VET.4	25	140.21	7	1.36	26.53	7.11	699.98
II	VET.1	n.d.	353.17	19	240.68	63.90	51.38	568.39
	VET.5	34	26.45	10	2.56	27.10	110.51	724.47
	VET.6	29	31.75	12	3.16	16.76	159.74	724.28
	VET.7	18	6.61	3	315.12	5.22	8.19	11.74
III	VET.2AA	n.d.	n.d.	6	1.38	23.18	8.96	680.83
	VET.3AA	n.d.	n.d.	7	1.37	20.77	8.29	572.56
	VET.4AA	n.d.	n.d.	5	1.50	21.46	8.79	584.11

n.d.—not determined; P.I.—plasticity index; A.I.—abrasivity index; C.E.C.—cation exchange capacity; E.C.—exchange cations.

**Table 5:** Main physical and technological properties of the studied samples (Cont.).

Group	Samples	C.K. (min)	O.A. (%)	pH	Exp. (%)	S.S.A. (m^2^/g)
I	VET.2	13.6	29	7.0	19.5	22.50
	VET.3	19.0	30	7.3	12.3	22.58
	VET.4	19.4	31	7.7	14.8	22.11
II	VET.1	18.4	43	6.8	17.8	13.75
	VET.5	37.8	37	7.3	13.6	42.55
	VET.6	30.1	37	7.7	10.9	44.71
	VET.7	30.3	63	8.6	3.1	5.09
III	VET.2AA	n.d.	n.d.	7.6	n.d.	n.d.
	VET.3AA	n.d.	n.d.	7.8	n.d.	n.d.
	VET.4AA	n.d.	n.d.	7.8	n.d.	n.d.

n.d.—not determined; C.K.—cooling kinetics; O.A.—oil absorption; Exp.—expandability, S.S.A.—specific surface area.

**Table 6:** Veterinary clay group characterizations.

Properties	VET.1	VET.2	VET.3	VET.4	VET.5	VET.6	VET.7
Adhesiveness [17,18,20,22,29,31]	▲	▲	▲	▲	▲	▲	●
Abrasiveness [17,18,20,21,22,29,31,32]	▼	▼	▼	▼	▲	▲	▲
Hazardous elements [20,23,28,31,32]	▼	▲	▲	▲	▼	▼	▼
Essential elements [20,21,30,31,32]	▲ Ca^2+^, Na^+^	▲ Ca^2+^	▲ Ca^2+^	▲ Ca^2+^	▲ Ca^2+^, K^+^	▲ Ca^2+^, K^+^	▲ Na^+^
Oil Absorption [18,20,21,22,31,32]	▲	▲	▲	▲	▲	▲	▲
Heat Retention [18,20,21,27,31,32]	▲	●	●	●	▲	▲	▲
Antibacterial performance [6,24,25,26]	▼	▲ VET.2AA	▲ VET.3AA	▲ VET.4AA	▼	▼	▼

▲ Advisable; ● Advisable with limitations; ▼ Needs vigilance.
